# 3′ IsomiR Species and DNA Contamination Influence Reliable Quantification of MicroRNAs by Stem-Loop Quantitative PCR

**DOI:** 10.1371/journal.pone.0106315

**Published:** 2014-08-29

**Authors:** Anita Schamberger, Tamás I. Orbán

**Affiliations:** 1 Institute of Enzymology, Research Centre for Natural Sciences, Hungarian Academy of Sciences, Budapest, Hungary; 2 Chemical Technology Transfer Ltd., Budapest, Hungary; Duke University, United States of America

## Abstract

MicroRNAs (miRNAs) are ∼20–24 nucleotide-long regulatory RNAs that have been proven to play important roles in many cellular processes. Since their discovery, a number of different techniques have been developed to detect and accurately quantify them. For individual mature miRNA measurements, quantitative stem-loop real-time PCR represents a widely used method. Although there are some data on optimization of this technique, there are still many factors that have not been investigated yet. In this study, we have thoroughly optimized this technique and pointed out several important factors that influence reliable quantification. First, we found that total RNA input can affect the measurements. Second, our data showed that carryover DNA contamination could also mislead the detection in a sequence-specific manner. Additionally, we provided evidence that different 3′ isomiR species of a particular miRNA can be reverse transcribed and cross-detected even by specifically targeted assays. Besides these, we have investigated the measurement of reaction efficiencies from total RNA samples and the accuracy of simultaneous reverse transcription reactions for increasing reliability and cost effectiveness without the loss of sensitivity and specificity. In summary, we provide a detailed, refined protocol for reliable detection of microRNA species by quantitative stem-loop PCR.

## Introduction

MicroRNAs (miRNAs) are short, non-coding regulatory RNA molecules that control mRNA stability and translation by targeting the 3′ untranslated region of given mRNA species [1], [2]. They influence various cellular functions and now are believed to form a crucial and extensive regulatory network similar to that of transcription factors [3]. The biogenesis of miRNAs consists of different, subsequent processing steps during which mature miRNA is liberated from longer precursor RNA forms [4]–[6]. In order to understand proper regulation and function, the different RNA forms can be studied and measured by various techniques. In the general laboratory practice, however, it is often sufficient to measure individual mature miRNA steady state levels. Nevertheless, measurements are challenging due to their short size, and sequence specific detection methods are more limited than in the case of mRNA molecules. Traditional hybridization techniques using radioactively or fluorescently labeled nucleic acids are generally applied, including in situ hybridization [7], [8] or Northern blotting [9]–[11]. Their sensitivity can be strongly increased by using specifically modified artificial nucleotides, such as locked nucleic acids (LNAs) [12]–[15], but miRNAs with low abundance can still be beyond the sensitivity of these methods [16], [17].

**Figure 1:**
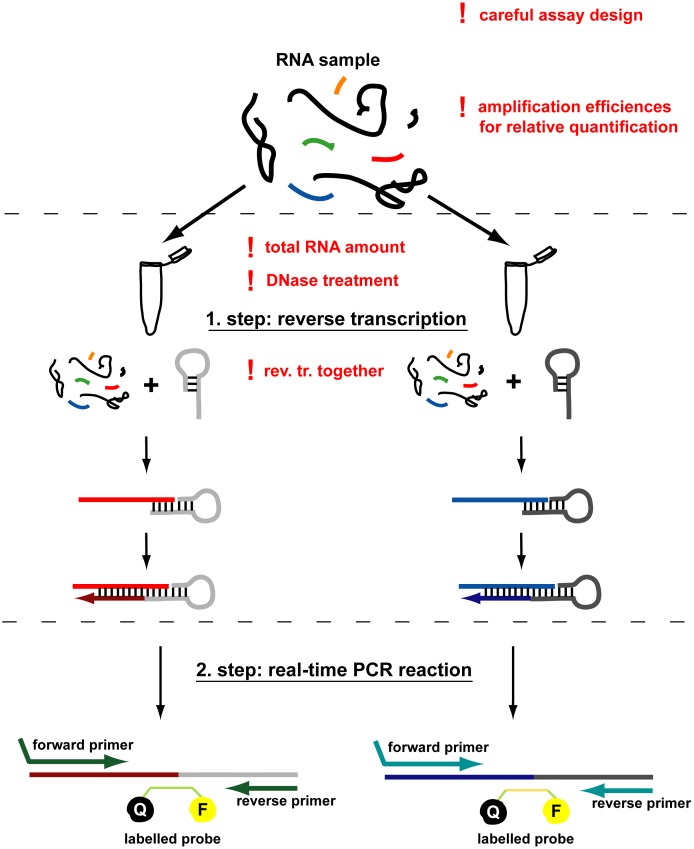
Schematic representation of stem-loop microRNA quantitative RT-PCR. The two main steps are reverse transcription and real-time PCR. In the first step, mature miRNA is extended and reverse transcribed by a sequence specific stem-loop primer. In the second step, the reverse transcribed miRNA is quantified by a fluorescently labeled hybridization probe using the strand replacement reaction. According to the previous protocol, all targets (e.g. endogenous control and target) should be reverse transcribed separately. In the dual-labeled probe based detection systems Q stands for quencher, F for fluorophore. Red exclamation marks indicate crucial points of the procedure that are discussed in this paper.

Similarly to mRNA detection and quantification, measuring the expression level of miRNA species by real-time PCR represents one of the most sensitive and accurate methods developed so far for such purposes. However, due to the short nature of miRNAs, a specific stem-loop real-time PCR technique has been developed among other methodologies [18]–[20]. The detection of mature miRNAs by this technique is composed of two main steps (Figure 1). The first step is a specifically targeted cDNA synthesis when a sequence specific stem-loop primer is hybridized to the mature miRNA and used to initiate the reverse transcription reaction. The second step is the real-time PCR during which the extended and transcribed miRNA is quantified using oligos specific for the miRNA and the primer loop sequences. This technique is fast and could be standardized for high-throughput purposes. However, this method has the *a priori* assumption that the miRNA in question has a well-defined 3′ end. Conversely, based on deep sequencing results, recent reports described significant sequence length heterogeneity of miRNAs originating from a given locus, often having significant variability of their 5′ and/or 3′ ends [21], [22]. Moreover, the distribution of such isomiRs seems to vary among cell types or physiological statuses of the cells [23], [24]. Therefore, such 3′ end variability could seriously influence miRNA detection by stem-loop PCR by interfering with the very first step, the sequence specific reverse transcription. There are several data on optimization of miRNA detection from discussing RNA isolation techniques to comparing various platforms [25]–[30]. Nevertheless, there are many other factors during individual mature miRNA detection by the widely used stem-loop quantitative PCR that are not discussed yet, although they play important roles in the accuracy and reproducibility of the measurements.

In this study, we intended to systematically investigate the stem-loop real-time PCR detection method of small RNA molecules. Careful optimization of this technique pointed to a previously underestimated aspect, that total RNA input and DNA contamination could severely influence the accurate detection. Moreover, we provide evidence that 3′ isomiR species are not exclusively measured by the stem-loop qRT-PCR methodology, and thereby can be cross-detected. This latter problem could not be overcome even by using the poly(A)-tailing-based qRT-PCR methodology. On the other hand, simultaneous reverse transcription of the target miRNA and the endogenous control does not necessarily influence the outcome of the results and may be a more accurate and cost effective approach for miRNA level quantitation. Based on our experiments, we suggest a refined protocol of miRNA detection by stem-loop real-time PCR technology.

## Results

### Relative quantification, reaction efficiency and the amount of reverse transcribed RNA

In quantitative RT-PCR applications, determination of the target is based on absolute or relative quantification. For individual miRNA measurements, relative quantification is the commonly used method, when the amount of the target is determined relative to an endogenous control. Since the target is compared to the control, they must be amplified with similar efficiencies. The accurate amplification efficiency in practice is calculated from the slope of a standard curve made by at least 5 points, encompassing the relevant concentration range of the application. Making an accurate and reproducible standard curve for miRNAs from total RNA samples (which is physiologically more relevant than using synthetic oligos) is challenging, since many miRNAs are present in low abundance. A sensitive balance has to be found between the sufficient dilution of the reverse transcription reaction (e.g.: for mRNA detection, it is a minimum of 1∶10) and an optimal C_t_ value (delayed by the dilution of the reverse transcription reaction; Figure S1). Therefore, we recommend the employment of small dilution steps (e.g. 1.5×) with only 3 or 4 points in the strict range of the measurement. If there is appropriate correlation between the control and target, the relative quantification method can be used at the particular dilution range for the analysis of the measurements.

**Figure 2:**
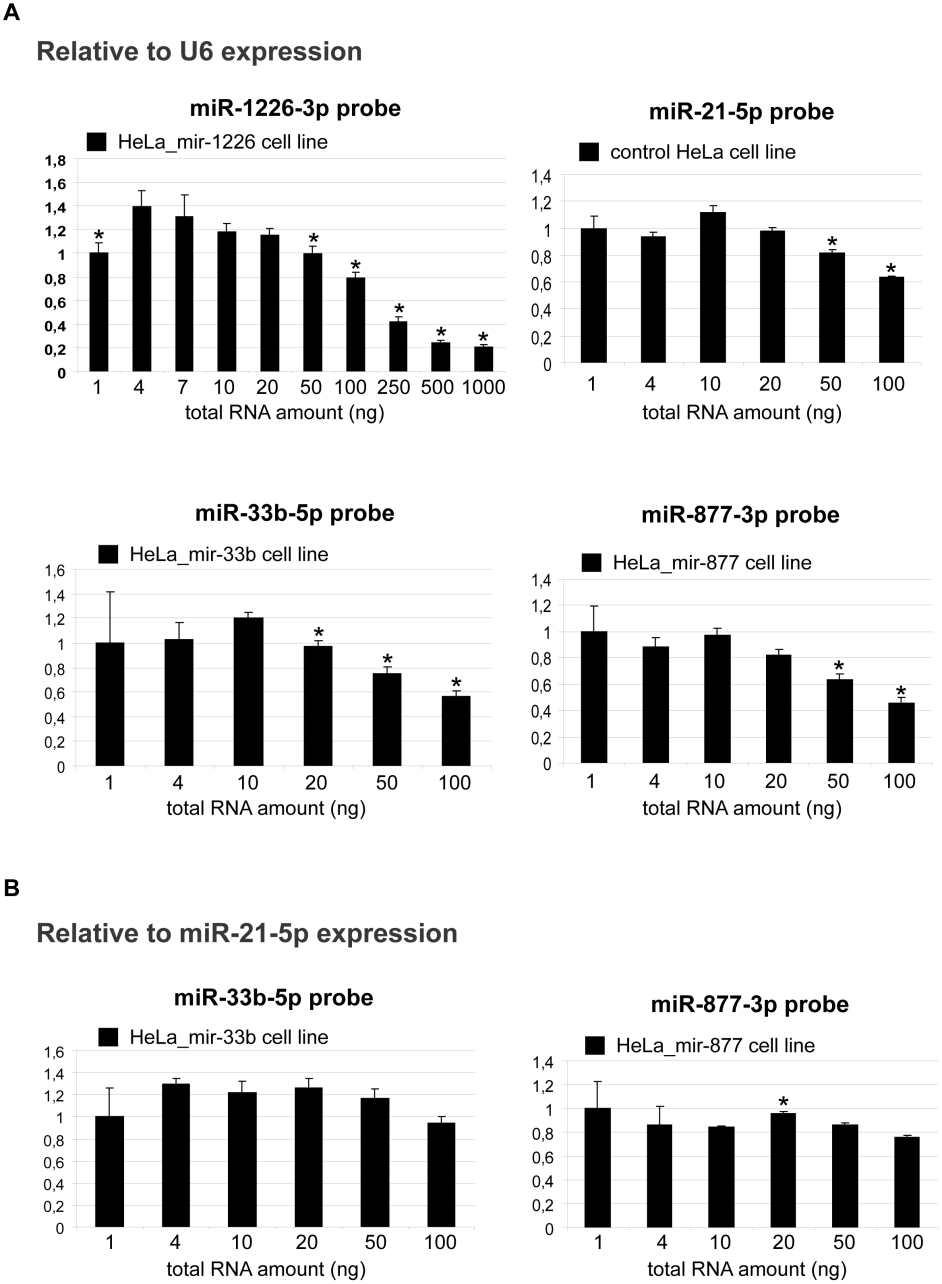
Mature miRNA detection from different amount of total RNA input. Different amount of total RNA samples were reverse transcribed for the detection of a particular mature miRNA by real-time PCR. MiRNAs with various abundance were measured, using relative quantification. The more abundant miR-1226-3p, miR-33b-5p and the less abundant miR-877-3p (mir-877*) were measured from cell lines stably overexpressing the corresponding miRNA, while the abundant endogenous miR-21-5p from parental HeLa cell line. Concerning the endogenous controls, the U6 snRNA (**A**) and the endogenous miR-21-5p (**B**) were applied. The optimal range of RNA input varies depending on endogenous controls and targets. The corresponding concentration series (controls and targets) were prepared and measured simultaneously. Mean values of three independent experiments (three biological parallels with three technical replicates) are shown. Error bars represent S.E.M.; samples are compared to a chosen optimal condition (10 ng of total RNA). *: p<0.05.

The next question is about the optimal amount of total RNA used for the reverse transcription reaction. As mentioned above, mature miRNA levels are often low in certain samples. Therefore, one could speculate to increase the amount of total RNA to increase the input of mature miRNAs in the reverse transcription reaction. To investigate this question, we measured more and less abundant miRNAs (abundance was estimated based on previous data: http://www3.appliedbiosystems.com/cms/groups/mcb_marketing/documents/generaldocuments/cms_089374.pdf and [17], [30]) from different total RNA input, relative to the widely used U6 small nuclear RNA (snRNA) or to the miR-21-5p endogenous miRNA. Target and endogenous control samples were prepared simultaneously and measured in the same plate during the real-time PCR reaction. The increase of the total RNA amount resulted in a decrease of mature miRNA detection when applying the U6 endogenous snRNA control (Figure 2A). It has dropped significantly above 20 ng in general and the effect did not seem to depend on the abundance of the miRNA target. Considering the miR-21-5p endogenous control, the effect of the total RNA input on the measurements was less pronounced (Figure 2B). Finally, the lower range of RNA input was measured less accurately probably because the low template concentration leads to delayed C_t_ values. Summarizing the results, the optimal range of RNA input varies depending on endogenous controls and targets, therefore, should be optimized. Based on our data, however, 10 ng total RNA input can be appropriate when using U6 endogenous snRNA control and 20 ng with the application of miR-21-5p endogenous miRNA control.

### Different targets can be reverse transcribed in the same reaction

**Figure 3:**
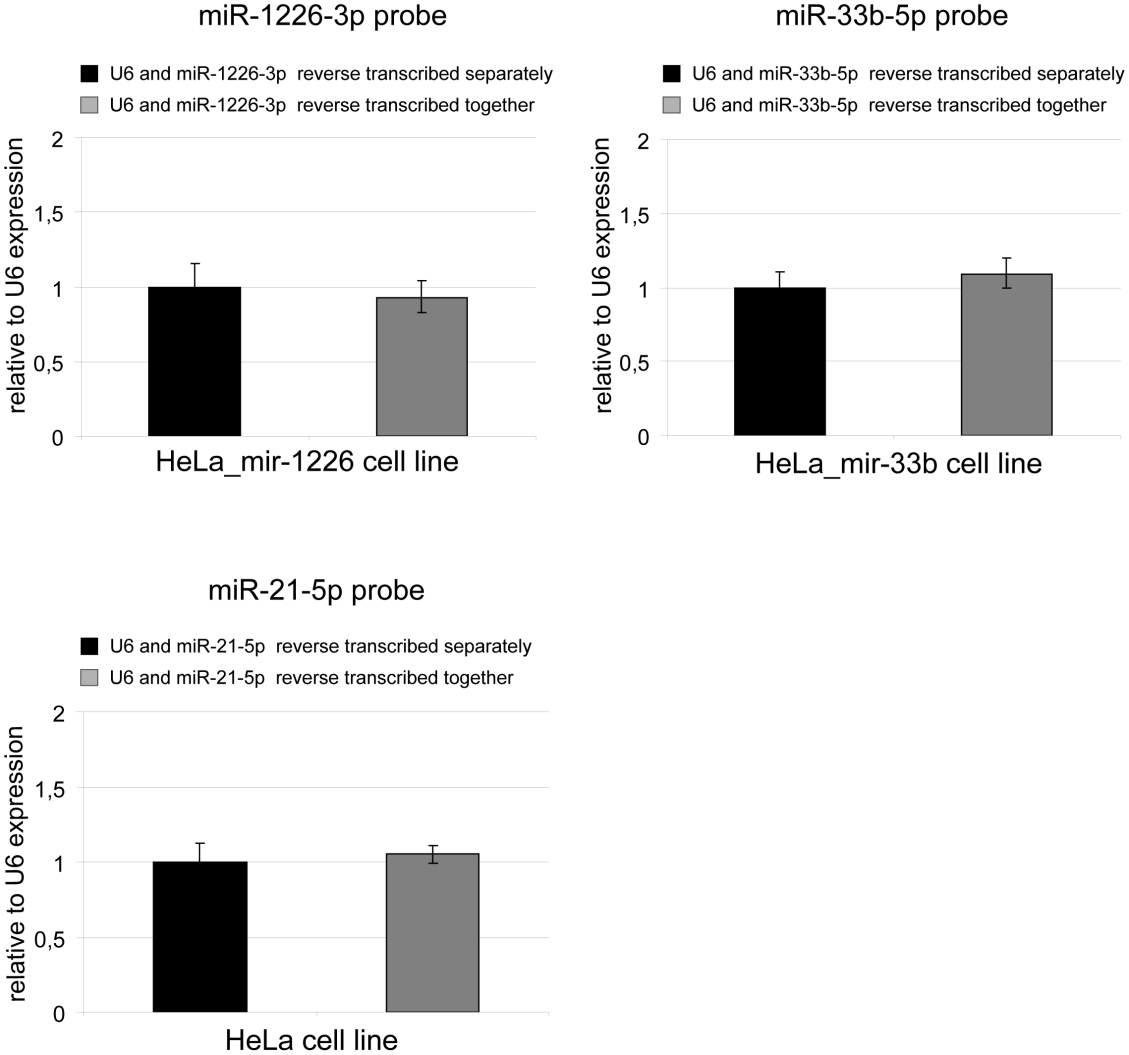
Reverse transcription of target and control can be done simultaneously. Mature miRNA levels of miR-1226-3p and miR-33b-5p were detected in stably overexpressing HeLa cell lines, whereas the endogenous miR-21-5p in parental HeLa cell line. cDNA samples were used from simultaneous or separate reverse transcription reactions of the endogenous control and the target. Experiments were carried out with three RT parallels and three technical replicates, error bars represent standard deviations.

For cDNA synthesis of miRNAs, the different small RNA targets have unique, sequence specific stem-loop primers to assist their reverse transcription. Although numerous miRNAs are reverse transcribed together in array experiments [31]–[33], it is indicated in the general protocol that for individual miRNA measurements, the endogenous control and the target have to be reverse transcribed in separate reactions (http://tools.lifetechnologies.com/content/sfs/manuals/cms_042167.pdf). To examine the feasibility of the simultaneous reactions, we compared real-time PCR measurements of simultaneously and separately reverse transcribed samples. We measured the level of several miRNAs including miR-1226-3p and miR-33b-5p in stably overexpressing HeLa cell lines, and the endogenous miR-21-5p in normal HeLa cell line. We found that there is no significant difference in the results when the reverse transcription was done separately or together with the endogenous control for the investigated assays (Figure 3). However, it is important to note that the long term storage of different hairpin primers mixed together in the same solution is not recommended as it may lead to a false positive detection of mature miRNAs (data not shown). In summary, the level of an individual miRNA can be determined by using cDNA samples in which the given target and the endogenous control are reverse transcribed together, thereby reducing potential pipetting errors and making the measurements more cost effective.

### DNA contamination significantly influences the measurement of mature miRNAs

**Figure 4:**
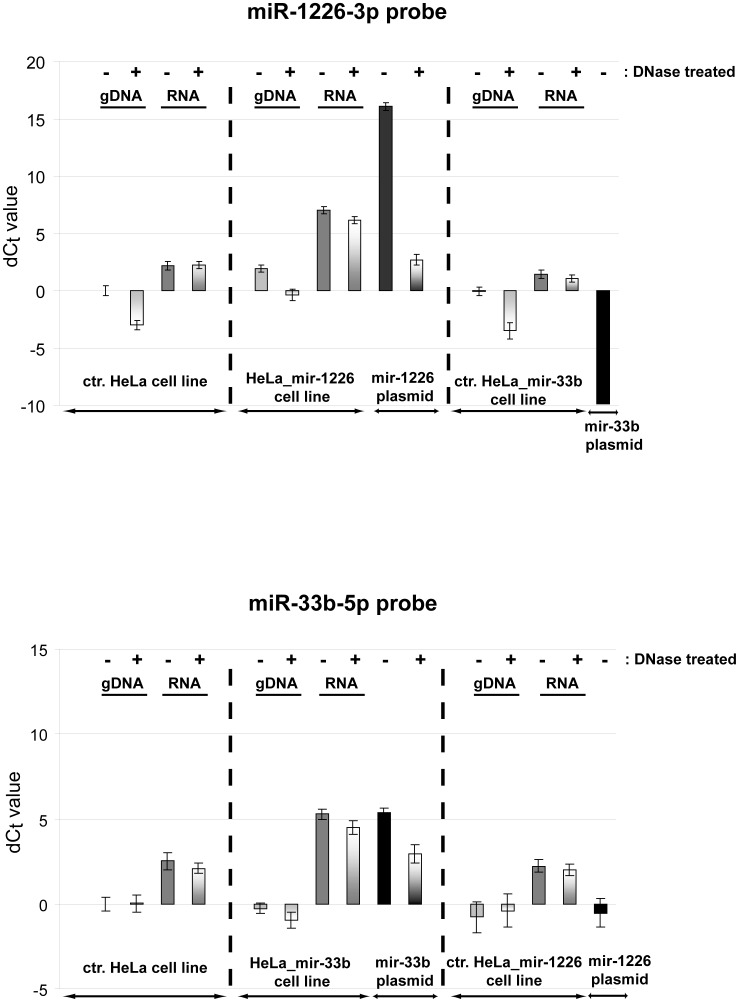
The effect of DNA contamination on mature miRNA measurements. Mature miR-1226-3p and miR-33b-5p detection were tested on the indicated samples, with or without DNase treatment. On the y-axis, dC_t_ value is represented, calculated as the Ct difference between the examined samples and the gDNA of control HeLa cell line (Ct = 33,8 for miR-1226-3p and Ct = 34,9 for miR-33b-5p experiments). Signal was not detected up to 40 reaction cycles for the mir-33b plasmid control by the miR-1226-3p assays. One C_t_ difference represents about 2× higher detected mature miRNA level. The effect of DNA is probe specific and plasmid DNAs have more pronounced effect on the measurements than gDNA contaminations. Experiments were carried out with three replicates at least twice, one representative experiment is shown. Error bars represent standard deviations.

Next, we investigated the effect of genomic and plasmid DNA on miRNA measurements. Based on our previous data from transient transfections, we had indications that contaminating DNA might interfere with mature miRNA detection. Thus, we examined whether stem-loop qRT-PCR is specific to the present mature miRNA or it has false positive signal from samples which do not contain the particular target. We tested miR-1226-3p and miR-33b-5p assays on genomic DNA (gDNA), total RNA and plasmid DNA (encoding the corresponding miRNA) samples. The investigated plasmids differ only in their pre-miRNA coding sequence. Total RNA and gDNA samples were derived from mir-1226 and mir-33b overexpressing or parental HeLa cell lines. We compared DNase treated and non-treated parallels for each sample. After reverse transcription, we measured the mature miRNA levels in the above samples (Figure 4). The miR-1226-3p probe detected 4 fold higher amounts (2 C_t_ difference) of mature miRNA from the gDNA of mir-1226 overexpressing cell line compared to the two “non-relevant” gDNA samples (from HeLa and HeLa_mir-33b cell lines). In the case of the miR-33b-5p assay, the measured miRNA levels were similar among all gDNA samples. For RNA samples, as it was expected, both miR-1226-3p and miR-33b-5p probes resulted in significantly higher detected mature miRNA levels from the corresponding miRNA overexpressing cell line than in the controls. Concerning plasmid DNAs, apparently similar amount of miR-33b-5p was detected from mir-33b encoding plasmid as from mir-33b overexpressing cell line derived RNA. This striking false effect was even more pronounced in the case of miR-1226-3p when the mir-1226 expression plasmid served as a template. There was 9 C_t_ difference compared to the mir-1226 overexpressing cell line derived RNA sample, and about 14 C_t_ difference compared to the RNA backgrounds, representing an apparent 512× and 16384× higher miRNA amount, respectively. From plasmids encoding other “non-relevant” miRNA, very low signals were detected both for miR-1226-3p and miR-33b-5p. The above data indicate that the false positive signals from the relevant plasmid samples are miRNA sequence specific. Therefore, although mature miRNA molecules are not present, signals can be apparently detected from DNA containing the coding sequence of the corresponding pre-miRNA form. These results were also confirmed by experiments using miR-877-3p and miR-877-5p assays (data not shown).

**Figure 5:**
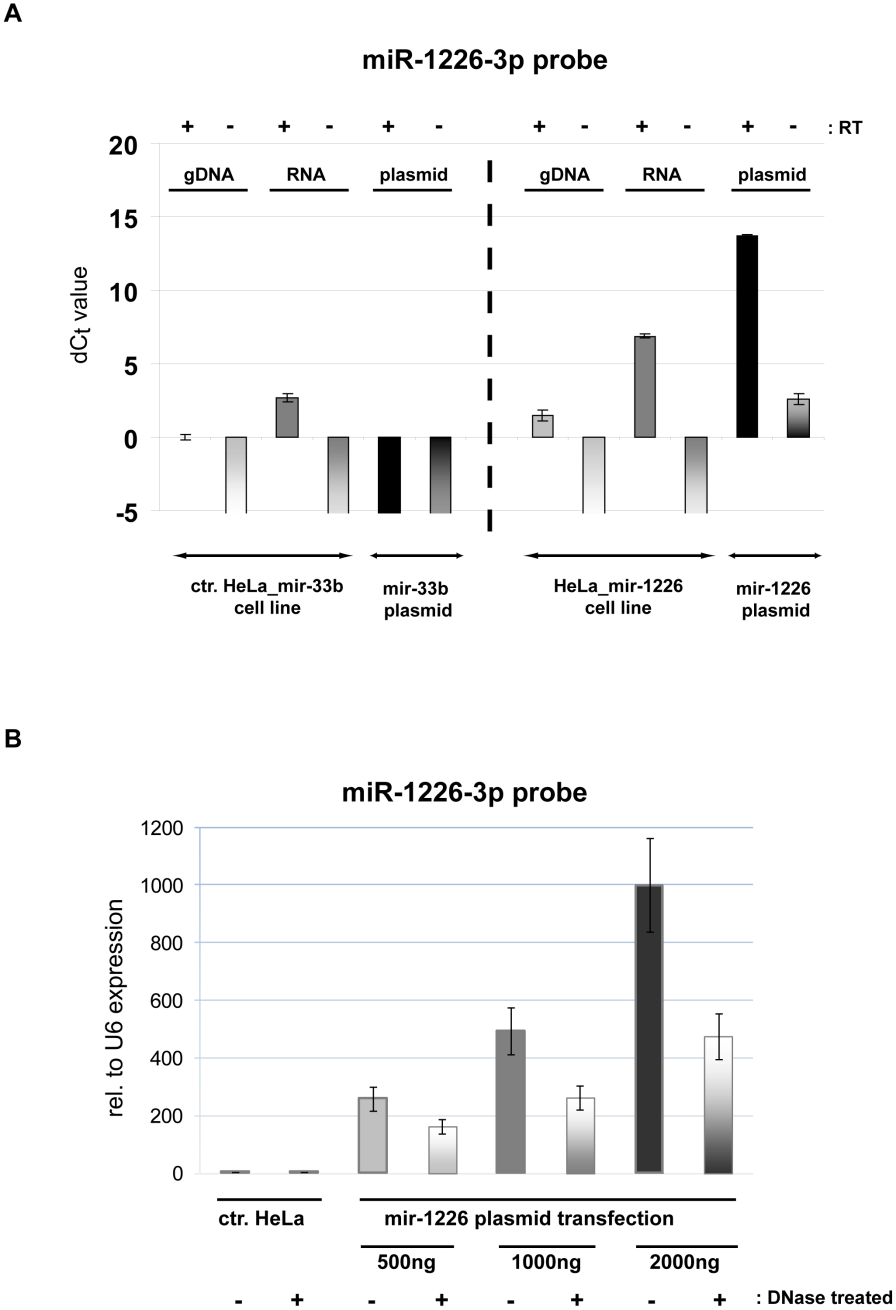
DNA can serve as a template during miRNA detection. (**A**) False positive signal of DNA derives mainly from the reverse transcription reaction. Mature miR-1226-3p was tested in the indicated samples, with or without reverse transcription (RT). On the y-axis, dC_t_ value is represented, calculated as the Ct difference between the examined samples and the gDNA of control HeLa_mir-33b cell line (Ct = 35,9). One C_t_ difference represents about 2× higher detected mature miRNA level. (**B**) DNA contamination remains in total RNA samples during isolation by the widely used Trizol reagent. Total RNA samples were isolated from transiently transfected HeLa cells; the transfected plasmid DNA amounts are indicated. Samples were DNase treated and non-treated, then reverse transcribed and subjected to real-time PCR. Expression values relative to U6 snRNA are shown on the y-axis. Experiments were carried out with three replicates at least from three independent experiments; one representative experiment is shown, error bars represent standard deviations.

Next, we intended to address the question that which part of the measurement (reverse transcription or real-time PCR) misleads the mature miRNA detection. To answer this question, first we made quantitative real-time PCR for miR-1226-3p from reverse transcribed and non-transcribed samples. We tested gDNA and RNA samples from mir-1226 overexpressing cell line and we also used mir-1226 encoding plasmid samples. Mir-33b overexpressing cell line and mir-33b encoding plasmid samples served as non-relevant controls. As shown in Figure 5A, there is a slight detection during the real-time PCR reaction from the relevant plasmid DNA without reverse transcription, but the majority of the false positive signal is detected only when the reverse transcription reaction is performed. We obtained similar results with the miR-33b-5p assay (Figure S2). In summary, these data reveal the unexpected fact that DNA may serve as a template during the reverse transcription reaction in a (stem-loop primer) sequence specific manner.

In further experiments, we tested whether the above phenomenon has a real relevance, for example when investigating miRNAs in transiently transfected cells. In such cases, RNA samples are prepared from cells containing the transfected plasmids. For these measurements we used RNA samples from HeLa cells transiently transfected with different amounts of a mir-1226 encoding plasmid (Figure 5B). The data showed that when samples were not treated with DNase, a significantly higher amount of miRNA was detected as compared to the DNase treated samples. This problem occurred not only by using the Trizol based total RNA isolation method, but also when applying a column-based isolation protocol such as the *mir*Vana Kit (Figure S3). These results indicate that there is plasmid DNA contamination in the total RNA samples which indeed misleads the accurate detection of mature miRNAs.

### 3′ isomiR forms of miRNAs are cross-detected

Emerging data strengthen the existence of isomiRs which are the results of the heterogeneous nature of miRNA processing, leading to variation in the length and/or sequence of mature miRNAs [23]. Since the exact 3′ end sequence seems to be crucial for stem-loop quantitative PCR, we investigated whether the different 3′ end variants of miRNAs can be exclusively detected by this technique. We applied different assays, designed for different 3′ isomiR species of a particular miRNA and tested the detection on various synthetic RNA oligonucleotides (∼10^5^ molecules/reaction).

**Figure 6:**
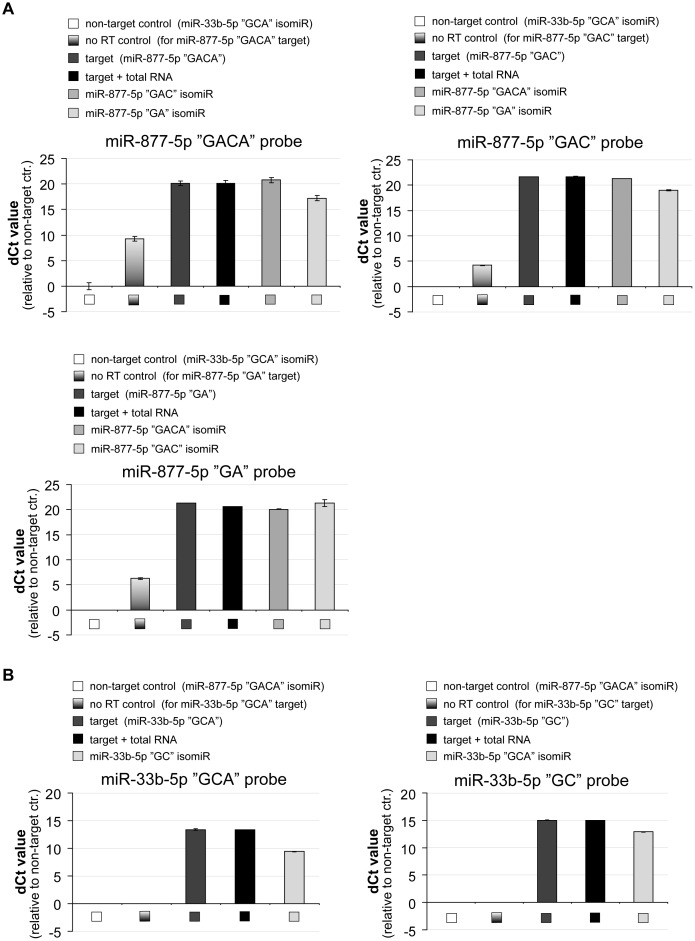
3′ isomiR species may not be distinguished by the stem-loop qRT-PCR. Detection of the 3′ isomiRs of miR-877-5p (**A**) and miR-33b-5p (**B**) by the corresponding stem-loop assays using synthetic RNA oligonucleotides as templates. Non-target controls were chosen as reference samples. (Ct values were 36/37/39,6 for the “GACA”/“GAC”/”GA” miR-877-5p isomiRs respectively, while signals were not detected up to 40 reaction cycles for the miR-33b isomiR assays.) The 3′ isomiRs of a particular miRNA locus are cross-detected using stem-loop qRT-PCR, although the extent varies among the different probes. Experiments were carried out at least in four independent measurements. One representative experiment is shown, error bars represent standard deviations.

First, among numerous 3′ isomiRs of miR-877-5p, the three most abundant species were analyzed by specific assays (http://www.mirbase.org/cgi-bin/get_read.pl?acc=MI0005561, at date of November, 2013). Since there were no commercially available pre-designed assays for them, we used custom made TaqMan assays (Life Technologies, CA, USA). They were tested on synthetic RNA oligonucleotides, identical to the miR-877-5p isomiR sequences. Each assay was tested for each isomiR species, bearing nucleotide differences in their 3′ ends (Figure 6A). Assays specific for the “GACA” and “GAC” 3′ends detected both “GACA” and “GAC” ended RNAs similarly, while “GA” ending was detected with ∼3 Ct delay. On the other hand, “GA” specific assay detected all three isoforms similarly. As concerning non-reverse transcribed (no RT) controls, signals were detected in the case of all three probes, indicating that these real-time PCR assays are somehow able to detect their synthetic RNA targets without reverse transcription. However, there were at least 10 Ct differences between the values of no RT controls and the reverse transcribed target containing samples.

In other experiments, we tested miR-33b-5p isomiRs. There are two indicated 3′ isomiR forms of miR-33b-5p in the miRBase database, with 1 nt difference in their 3′ ends. The shorter mature miRNA is marked as the reference sequence but the longer form seems to be more abundant in investigated cell lines based on deep sequencing data (http://www.mirbase.org/cgi-bin/get_read.pl?acc=MI0003646, at date of November, 2013). We tested these variants by commercially available pre-designed TaqMan assays (Life Technologies, CA, USA). Both “GCA” and “GC” 3′ end specific assays detected the corresponding template better than the other isomiR, but the cross-detections were still considerable (2–4 C_t_ delayed; Figure 6B). To examine if a different miRNA detecting qRT-PCR approach might overcome this problem, we analyzed the above miR-33b-5p isomiRs by using the poly(A)-tailing-based method [19]. However, the isomiRs were also strongly cross-detected in those experiments (Figure S4), and even the melting curve analysis could not make reliable indication that more isomiRs are present when applying mixed isomiR population as a template for the different assays (data not shown).

The above data indicate that the examined quantitative real-time PCR methods for miRNA detection are not exclusively specific for a given isomiR, consequently 3′ isomiR species can be cross-detected in various extent. These results underline that careful selection of the assay is essential, since the accurate measurement of the given mature miRNA species strongly relies on the selected assay.

## Discussion

In this study, we examined several factors in detail influencing accuracy and reliability of the miRNA quantitative stem-loop PCR. Considering the reverse transcription step of this methodology, our data indicate that the increase of the total RNA amount can result in a lower apparent miRNA expression level. This phenomenon could occur due to dissimilar reaction efficiencies of the target and the control in certain ranges of total RNA amount. Thus, it may lead to elevated detection of the endogenous control compared to the target (at higher concentration ranges, as suggested by the analysis of the raw data), therefore resulting in an apparent decrease in the level of the target. For a particular endogenous control/target pair the optimal amount of the total RNA for the reverse transcription reaction can vary, therefore pilot investigations are advisable prior to the real experiments. However, based on our experiments, 10–20 ng of total RNA might be adequate. In addition to these data, we provided evidence that the target of interest can be reverse transcribed together in one reaction with the appropriate endogenous control. Apart from lowering the costs of experiments, it has the advantage of reducing pipetting errors and thereby making the measurements more accurate.

Next, we found the unexpected result that contrary to the claims of the original protocol [18], DNA could serve as a template during mature miRNA measurements, mostly during the reverse transcription reaction. Our data suggest that the corresponding pre-miRNA coding sequence is detected by the stem-loop primer. There are data that reverse transcriptases can use (single stranded) DNA as a template, therefore this DNA dependent DNA polymerase activity might be an explanation for our observation. However, the extent of the DNA-derived false detection varied among different miRNA targeting assays, and plasmid DNAs had more pronounced effects on the detection than gDNA contaminations. The significance of DNA contamination is further underlined by the fact that miRNA expression studies are often carried out on transiently transfected cells, which contain a significant amount of plasmid DNA originating from the used expression vector. Additionally to this, DNA and RNA molecules are both detected at 260 nm by spectrophotometry, therefore DNA contamination also disturbs the accurate measurement of RNA concentration. All these factors imply that extensive DNase treatment is a critical part of this miRNA quantification protocol which cannot be omitted when using certain RNA isolation methods. Our data show, that in contrast to total RNA isolation using either Trizol reagent or *mir*Vana Kit, no significant DNA contamination present in the RNA samples when applying small RNA isolation by the *mir*Vana Kit (Figure S3).

In addition to the technical issues described above, the recently discovered isomiR species impose another challenge on miRNA detection by the stem-loop qPCR technique, as the sequence diversity of miRNA species could be quite extensive both at the 5′ and the 3′ ends. Although there are emerging data on the existence of this variability, neither all mechanisms responsible for the generation of isomiRs nor their potential functional differences are clear as yet. Even if 3′ variability appears to be redundant in function at present [23], it represents a problematic issue not only for stem-loop qRT-PCR, but also for miRNA detection by the poly(A)-tailing based methodology ([21]; Figure 6 and Figure S4). Therefore, the *a priori* knowledge of the exact 3′ sequence is a prerequisite for designing an accurate, specific assay for any particular small RNA species, and examining miRNA databases and available online deep sequencing data is strongly recommended. Additionally, we would like to point out that the indicated reference sequences in databases often represent only a small proportion of isomiRs, therefore it could mislead researchers in assay design. Thus, as it was shown in the case of miR-33b-5p and miR-877-5p, cautious selection or design of the assay is essential, since only one nucleotide difference in the 3′ end terminus can cause inaccurate detection, leading to false representation of a mature miRNA form.

In addition to our findings, we would like to note that besides the factors investigated here, there are other issues reported to influence reliable miRNA detection. For example, when applying the widely used Trizol reagent based RNA isolation method also for miRNAs, it is important to keep in mind that the extraction efficiency of miRNAs with low GC content or stable secondary structure is sensitive for the initial number of the cells [34].

Summarizing our results, we provide a detailed and improved protocol for proper application of quantitative stem-loop RT-PCR for the accurate detection of mature miRNA species (see Figure 1 and Materials and Methods).

## Materials and Methods

### Refined, detailed protocol for stem-loop quantitative RT-PCR of individual miRNAs

#### Assay design

Careful assay selection for the proper isomiR species is crucial to evade misleading data. If the desired isomiR species is not known for a given miRNA, several previously annotated abundant isoforms should be tested in parallel.

#### RNA isolation

If total RNA isolation is done by Trizol reagent, the usage of minimum 1–2×10^6^ cell/ml Trizol is strongly recommended (see ref [34]). The assessment of the quality of the isolated RNA sample (e.g.: by BioAnalyzer, Agilent Technologies) is also advisable.

#### DNase treatment (strongly recommended for total RNA samples)

e.g. 5 µg of total RNA,

2 µl (4 unit) of DNase (New England Biolabs),

2 µl of 10× DNase buffer,

1 µl (40 unit) of RNasin (Life Technologies), in total volume of 20 µl.

Incubate at 37°C for 1 hour, inactivate at 75°C for 10 minutes, then put on ice. Quantification of RNA by spectrophotometry (e.g.: NanoDrop 2000 Spectrophotometer, Thermo Scientific).

#### cDNA preparation (TaqMan MicroRNA Reverse Transcription Kit, Life Technologies)

For one reaction:

0.15 µl of 100 mM dNTP Mix,

1 µl of Reverse Transcriptase,

1.5 µl of 10× buffer,

0.19 µl of RNase inhibitor,

1.16 µl of H_2_O.

Mix gently, then add 5 µl of total RNA (2 ng/µl).

Mix gently and add 3 µl of endogenous control specific RT primer and 3 µl of target specific RT primer.

Reverse transcribe the RNA according to the manufacturer’s instructions (16°C for 30′, 42°C for 30′, 85°C 5′).

Important note: reverse transcription efficiency may vary among samples in different type of PCR tubes.

#### cDNA dilution for quantitative PCR

Dilute the total 15 µl of cDNA volume 5 times by adding 60 µl of H_2_O.

#### Quantitative real-time PCR (using TaqMan MicroRNA Assays, Life Technologies)

Perform the samples in triplicate in singleplex reactions, in a final volume of 20 µl.

for one reaction:

10 µl of 2× Mix (TaqMan Universal Master Mix II with UNG, Life Technologies),

1 µl of 20× probe,

9 µl of diluted cDNA.

The final dilution of the cDNA in the reaction is 11×. Always apply non-template controls for the different assays. Perform the PCR reaction according to the manufacturer’s instructions (50°C for 2′, 95°C for 10′, in 40 cycles: 95°C for 15″, 60°C 1′).

#### Data analysis

If relative quantification is to be applied, make sure by standard curve analysis that it is indeed applicable for comparison of the particular assays. Always check the baseline and threshold values since big differences in Ct values of the samples or little contamination in the non-template control might cause false auto fit by the program.

### Plasmid constructs and isolation

EGFP embedded mir-1226, mir-33b and mir-877 expression plasmids were cloned as described earlier [17]. Plasmid DNAs were isolated by QIAGEN Plasmid Midi Kit using EndoFree Plasmid Buffer Set.

### Cell cultures and manipulation

Parental HeLa cell line [35] was kindly provided by Zsuzsanna Izsvák (Mobile DNA Group, Max-Delbrück Center, Berlin, Germany). Cells were maintained in Dulbecco’s modified Eagle’s medium (DMEM) supplemented with 10% of fetal calf serum, 1% of L-glutamine, and 1% of penicillin/streptomycin (Life Technologies) using standard cell culture methodology. Mir-1226, mir-33b and mir-877 stably expressing cell lines were established by the *Sleeping Beauty* transposon based gene delivery technology as described earlier [17]. For transient transfections, 3×10^5^ HeLa cells per wells were seeded onto a 6-well plate for transfection on the next day by FuGENE HD reagent (Life Technologies) using plasmid DNAs as indicated (DNA:lipid reagent = 1 µg:3 µl). Transfection efficiencies were followed by EGFP fluorescence, detected by a IX51 fluorescence microscope (Olympus). Cells were collected for total RNA isolation 48 h after transfection.

### Genomic DNA isolation

After trypsinization, cells were centrifuged and washed with 1× phosphate-buffered saline. Then, after careful removal of the liquid supernatant, cell pellets were stored at −80°C until further processing. Genomic DNAs were isolated from the cells by standard phenol-chloroform extraction after cell lysis and proteinase K digestion. To remove RNA contamination from genomic DNA, samples were RNaseA treated at 37°C for 1 hour before proteinase K treatment.

### miRNA analysis

Total RNA was isolated from cultured cells using either the Trizol reagent or the *mir*Vana miRNA Isolation Kit (Life Technologies); small RNA samples were isolated using the *mir*Vana miRNA Isolation Kit (Life Technologies). ∼2×10^6^ of cells were harvested and prepared according to the manufacturer’s instructions. To remove DNA contaminations, RNA samples were treated with DNaseI (New England Biolabs) at 37°C for 1 hour. When applying the stem-loop qRT-PCR for cDNA preparations, if not indicated otherwise, 10 ng of total RNA (or gDNA, or plasmid) was reverse transcribed with miRNA specific stem-loop primers using TaqMan MicroRNA Reverse Transcription Kit (Life Technologies). For the poly(A)-tailing based qRT-PCR method, the miRCURY LNA Universal RT microRNA PCR Starter Kit from Exiqon was used following the manufacturer’s protocol. “no RT” controls were prepared by inactivating the reverse transcriptase at 98°C for 20 minutes prior to adding it to the cDNA Mix (stem-loop qRT-PCR) or leaving out the enzyme mix from the reaction (Exiqon Kit). Related sample series were prepared simultaneously and measured in the same plate during the real-time PCR reaction. The general “cutoff” value was 40 cycles, as a standard used by the program of the used instruments. We always used three technical replicates for the real-time PCR measurements, and biological and RT parallels as indicated in the figure legends. When measuring miR-33b-5p and miR-877-3p with different total RNA input (shown in Figure2A and B), we used the same three independent RNA samples (isolated from the respective miRNA overexpressing HeLa cell line) for the experiments with the U6 and miR-21-5p endogenous controls. For isomiR detections, synthetic 5′-phosphate RNA oligos were purchased from Sigma. Assuming an average of 10^5^ miRNA copies per 10 ng of total RNA [30], diluted RNA oligos were used either alone or supplemented with 10 ng of control HeLa total RNA samples for reverse transcription. Quantification was performed by quantitative real-time PCR using either TaqMan MicroRNA Assays and TaqMan Universal Master Mix II with UNG (Life Technologies) or LNA PCR primers sets and ExiLENT SYBR Green master mix (Exiqon). The real-time quantification reactions were performed on StepOne™ or StepOnePlus™ platforms (Life Technologies), according to the manufacturer’s instructions; the data was analyzed by StepOne software (version 2.1; Life Technologies). Our data were represented as by the StepOne program (relative to an endogenous control, delta delta Ct values) or when it was not relevant, as delta Ct values (comparing to a control sample). For statistical analysis, two-sided Student’s t-test was performed. The following TaqMan MicroRNA Assays were used in miRNA quantification, catalog numbers are in brackets: U6 small nuclear RNA [001973], hsa-miR-1226-3p [245467_mat], hsa-miR-33b-5p = hsa-miR-33b-5p “GC” [002085], hsa-miR-33b-5p “GCA” [001565], hsa-miR-21-5p [000397] and hsa-miR-877-3p = hsa-miR-877* [241029_mat]. For custom made miRNA assays, the following RNA sequences were used for assay design: 5′-GUAGAGGAGAUGGCGCAGGGGACA for the hsa-miR-877-5p “GACA” isomiR ( = hsa-miR-877-5p), 5′-GUAGAGGAGAUGGCGCAGGGGAC for the hsa-miR-877-5p “GAC” isomiR and 5′-GUAGAGGAGAUGGCGCAGGGGA for the hsa-miR-877-5p “GA” isomiR. For Exiqon LNA PCR primers sets, the following assays were used, catalog numbers are in brackets: has-miR-33b-5p “GC” [205860] and custom designed mir-33b-5p-GCA_1 “GCA” [206999].

## Supporting Information

Figure S1 **Determination of reaction efficiencies of different targets.** (**A**) Standard curves with 1.5× dilution series and 5 points. Template concentrations are presented in a logarithmic scale; R^2^ values represent the correlation coefficients of the fitted lines. (**B**) Amplification efficiencies calculated from different ranges of the curves. 1–5 for five points; 2–5 for four points, omitting the obvious outlier of the measurement from the most concentrated template. (It is below the recommended minimum of 1∶10 dilution of the cDNA sample in the qPCR reaction).
(TIF)

Figure S2 **DNA can serve as a template for the reverse transcription reaction.** False positive signal of DNA derives from the reverse transcription reaction. Mature miR-33b-5p assay was measured in the indicated samples, with or without reverse transcription (RT). On the y-axis, dC_t_ value is represented (calculated as the Ct difference between the examined samples and the gDNA of control HeLa_mir-1226 cell line). Control gDNA data are above C_t_ of 35; one C_t_ difference represents about 2× higher detected mature miRNA level. Experiments were carried out in three replicates; one representative experiment is shown, error bars represent standard deviations.
(TIF)

Figure S3 **Residing DNA contamination in RNA samples prepared by different RNA isolation procedures by** ***mir*** **Vana miRNA Isolation Kit.** RNA samples were isolated from parental (control) and transiently transfected HeLa cells. Samples were DNase treated and non-treated, then reverse transcribed and subjected to real-time PCR. Expression values relative to U6 snRNA are shown on the y-axis. Experiments were carried out with three technical replicates from three independent experiments (biol. repl.), error bars represent standard deviations. There is remaining DNA contamination in the total RNA samples (**A**), but not in the small RNA enriched samples (**B**) when prepared by the *mir*Vana Kit. The expression level of miR-1226-3p from total RNA (with DNase treatment) and from small RNA samples (with or without DNase treatment) is similar.
(TIF)

Figure S4 **3′ isomiRs of miR-33b-5p are cross-detected using the poly(A)-tailing based quantitative RT-PCR method.** 3′ isomiRs of miR-33b-5p were detected by isomiR-specific primer sets using synthetic RNA oligonucleotides as templates. Non-target controls served as references samples (Ct values >33). In the no RT control reactions, particularly no signals (Ct >39) were detected. The two different 3′ isomiRs are significantly cross-detected by the specific primer sets and even the post-PCR SYBR Green-based melting curve analysis could not make reliable distinction between the different isomiR-specific PCR products when applying mixed isomiR population as a template (data not shown). Experiments were carried out at least twice, one representative experiment is shown, error bars represent standard deviations.
(TIF)
