# Supplementary figures and images for: 3′ IsomiR Species and DNA Contamination Influence Reliable Quantification of MicroRNAs by Stem-Loop Quantitative PCR
Source: PLoS One. 2014 Aug 29;9(8):e106315. doi: 10.1371/journal.pone.0106315 (PMC4149544; doi:10.1371/journal.pone.0106315)

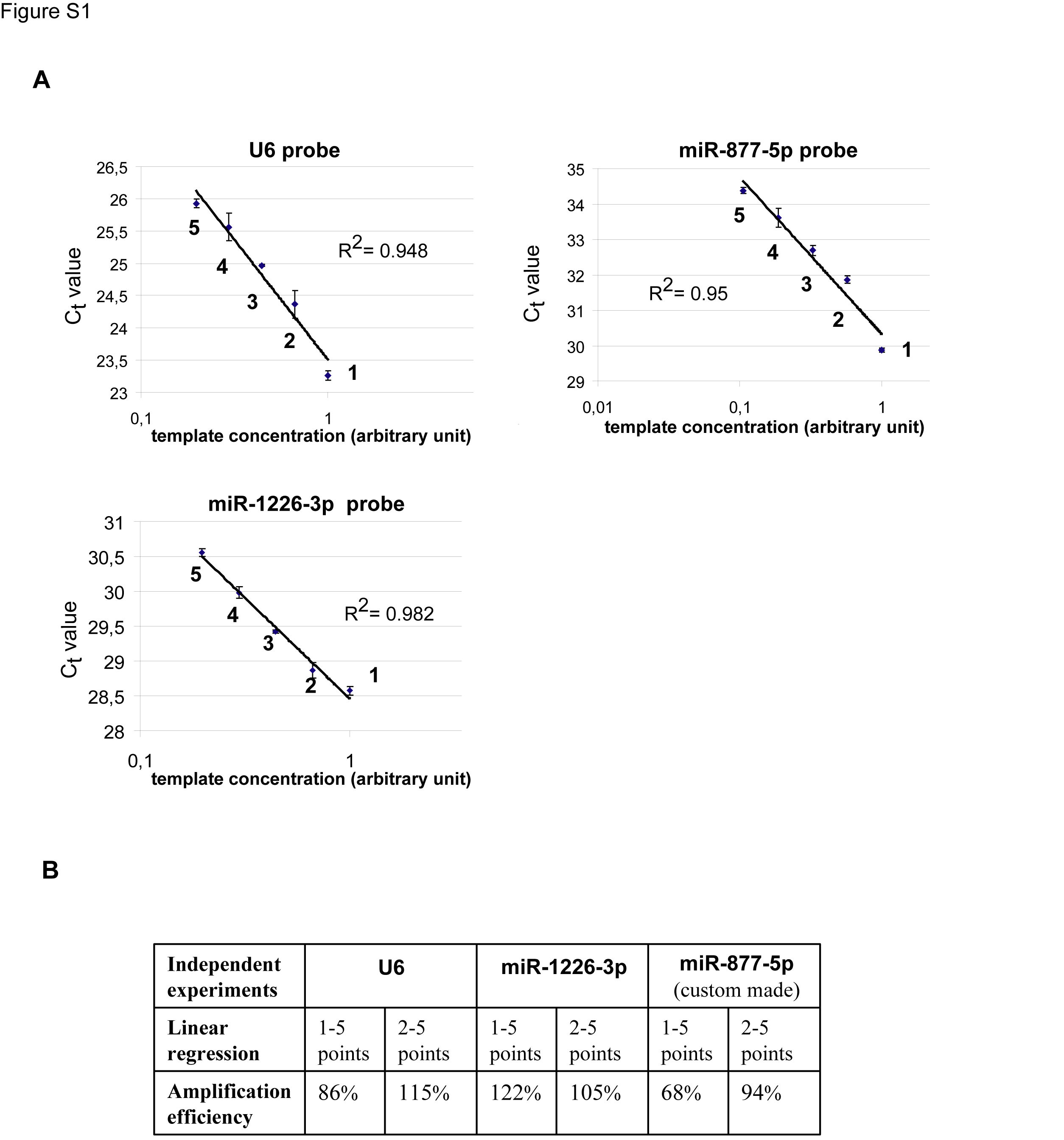

Supplement: Figure S1 — Determination of reaction efficiencies of different targets. (A) Standard curves with 1.5× dilution series and 5 points. Template concentrations are presented in a logarithmic scale; R2 values represent the correlation coefficients of the fitted lines. (B) Amplification efficiencies calculated from different ranges of the curves. 1–5 for five points; 2–5 for four points, omitting the obvious outlier of the measurement from the most concentrated template. (It is below the recommended minimum of 1∶10 dilution of the cDNA sample in the qPCR reaction). (TIF) [file pone.0106315.s001.tif]

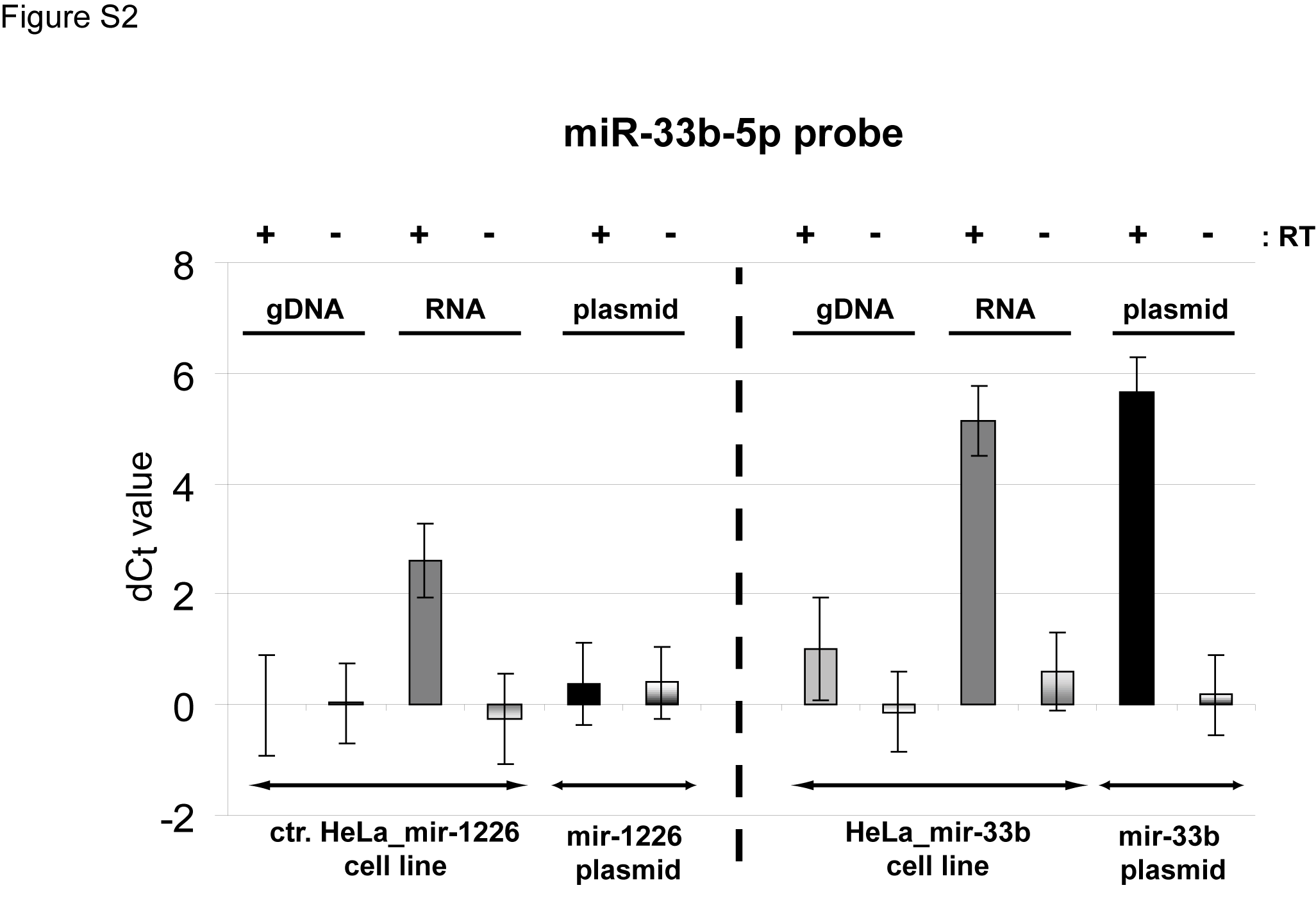

Supplement: Figure S2 — DNA can serve as a template for the reverse transcription reaction. False positive signal of DNA derives from the reverse transcription reaction. Mature miR-33b-5p assay was measured in the indicated samples, with or without reverse transcription (RT). On the y-axis, dCt value is represented (calculated as the Ct difference between the examined samples and the gDNA of control HeLa_mir-1226 cell line). Control gDNA data are above Ct of 35; one Ct difference represents about 2× higher detected mature miRNA level. Experiments were carried out in three replicates; one representative experiment is shown, error bars represent standard deviations. (TIF) [file pone.0106315.s002.tif]

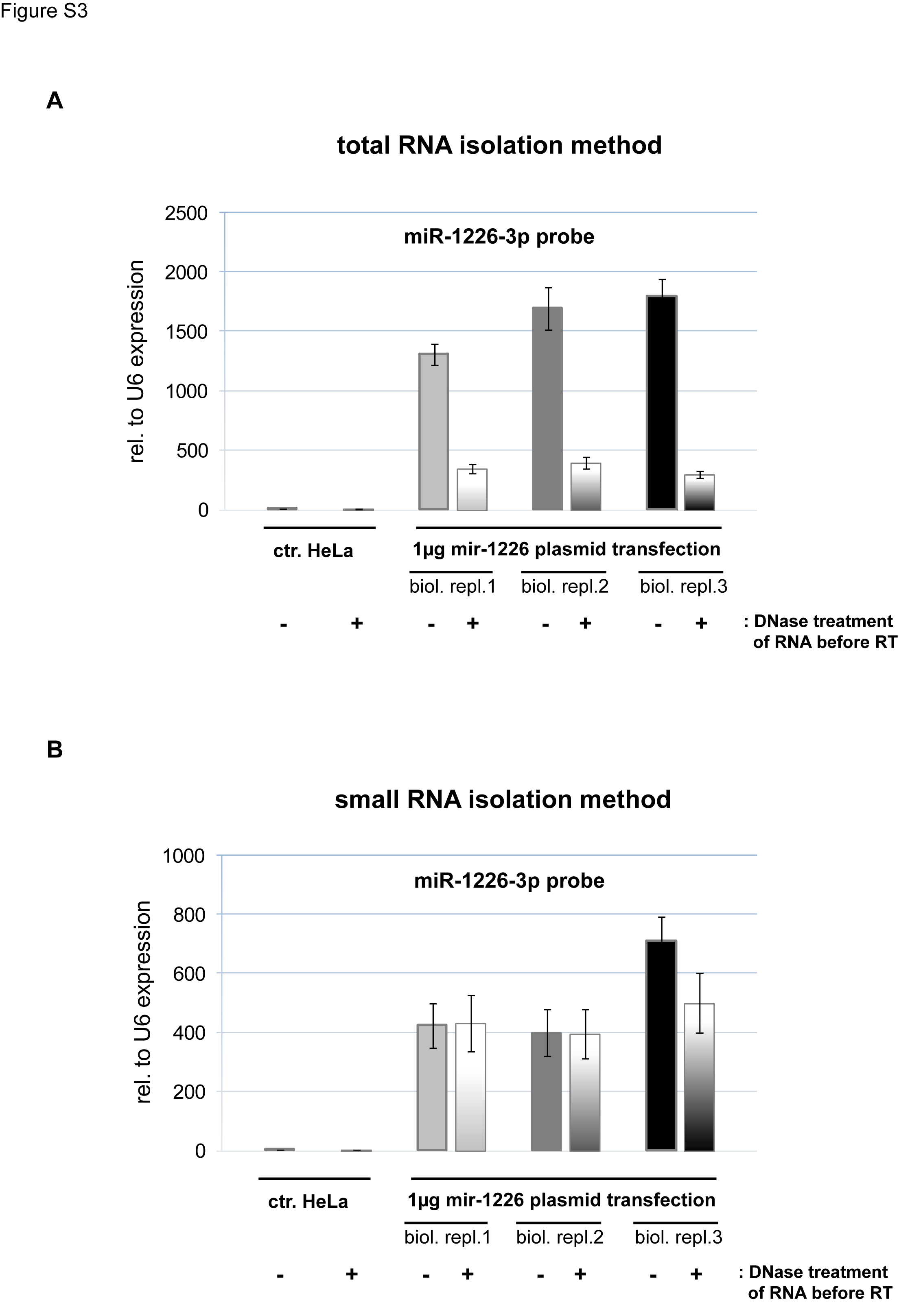

Supplement: Figure S3 — Residing DNA contamination in RNA samples prepared by different RNA isolation procedures by mir Vana miRNA Isolation Kit. RNA samples were isolated from parental (control) and transiently transfected HeLa cells. Samples were DNase treated and non-treated, then reverse transcribed and subjected to real-time PCR. Expression values relative to U6 snRNA are shown on the y-axis. Experiments were carried out with three technical replicates from three independent experiments (biol. repl.), error bars represent standard deviations. There is remaining DNA contamination in the total RNA samples (A), but not in the small RNA enriched samples (B) when prepared by the mirVana Kit. The expression level of miR-1226-3p from total RNA (with DNase treatment) and from small RNA samples (with or without DNase treatment) is similar. (TIF) [file pone.0106315.s003.tif]

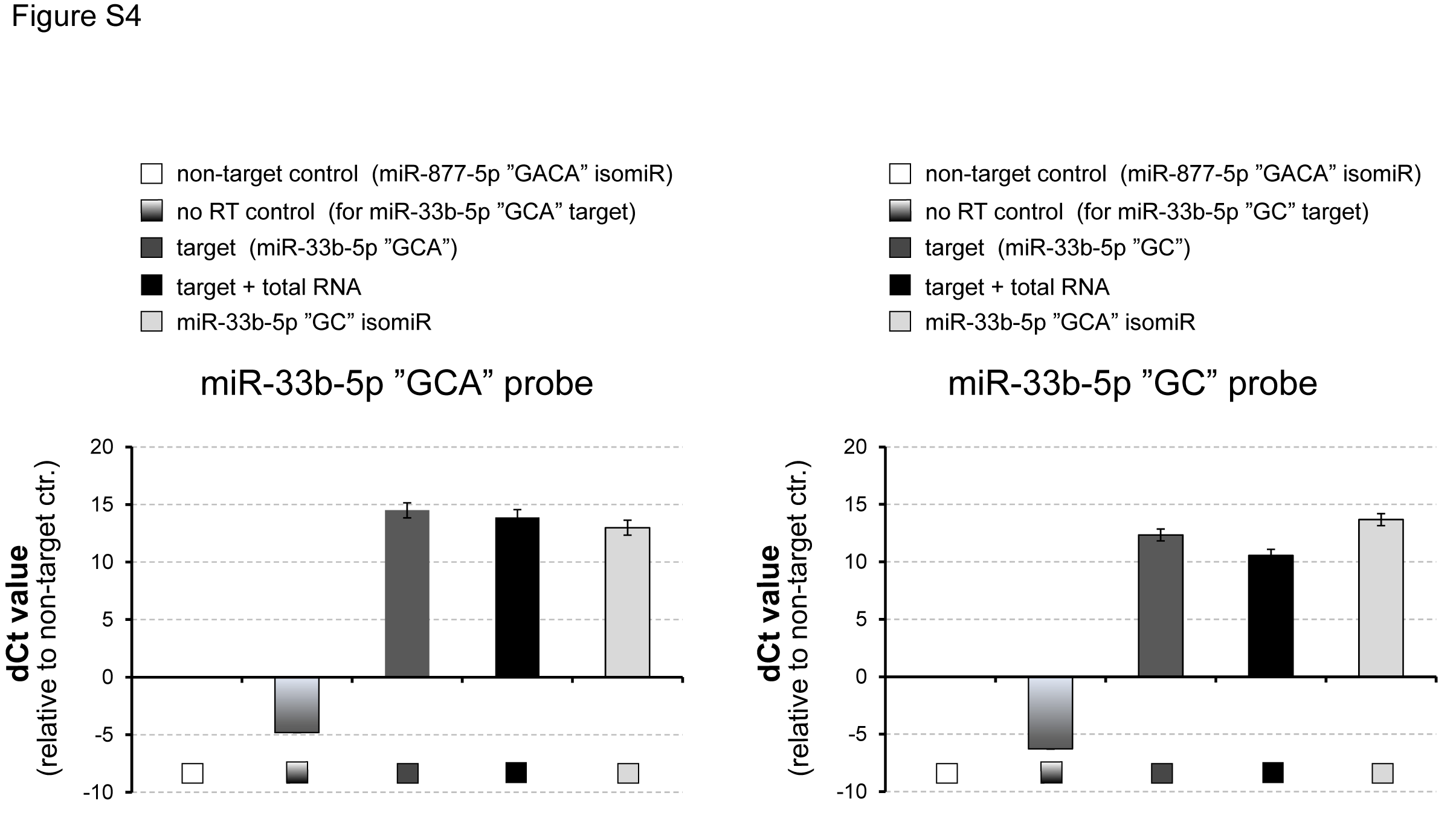

Supplement: Figure S4 — 3′ isomiRs of miR-33b-5p are cross-detected using the poly(A)-tailing based quantitative RT-PCR method. 3′ isomiRs of miR-33b-5p were detected by isomiR-specific primer sets using synthetic RNA oligonucleotides as templates. Non-target controls served as references samples (Ct values >33). In the no RT control reactions, particularly no signals (Ct >39) were detected. The two different 3′ isomiRs are significantly cross-detected by the specific primer sets and even the post-PCR SYBR Green-based melting curve analysis could not make reliable distinction between the different isomiR-specific PCR products when applying mixed isomiR population as a template (data not shown). Experiments were carried out at least twice, one representative experiment is shown, error bars represent standard deviations. (TIF) [file pone.0106315.s004.tif]
